# Stellettin B Isolated from *Stelletta* Sp. Reduces Migration and Invasion of Hepatocellular Carcinoma Cells through Reducing Activation of the MAPKs and FAK/PI3K/AKT/mTOR Signaling Pathways

**DOI:** 10.1155/2022/4416611

**Published:** 2022-11-29

**Authors:** Tsung-Chang Tsai, Wen-Tung Wu, Jen-Jie Lin, Jui-Hsin Su, Yu-Jen Wu

**Affiliations:** ^1^Department of Nephrology, Antai Medical Care Corporation Antai Tian-Sheng Memorial Hospital, Pingtung 92842, Taiwan; ^2^Department of Nursing, Meiho University, Pingtung 91202, Taiwan; ^3^Department of Food Science and Nutrition, Meiho University, Pingtung 91202, Taiwan; ^4^Yu Jun Biotechnology Co., Ltd., Kaohsiung 81363, Taiwan; ^5^National Museum of Marine Biology & Aquarium, Pingtung 94450, Taiwan; ^6^Frontier Center for Ocean Science and Technology, National Sun Yat-sen University, Kaohsiung 80424, Taiwan

## Abstract

Hepatocellular carcinoma (HCC) is one of the most common malignant tumors, and there is currently a lack of effective treatment options to control the metastasis. This study was performed to examine the mechanisms of the migration and invasion characteristics of HCC, with the aim of reducing metastasis by inhibiting cancer cell migration and invasion. In this study, we used Stellettin B, an active compound isolated from Stelletta sponges, as the experimental drug and evaluated its inhibition effects on cell migration and invasion in human hepatoma cells (HA22T and HepG2). MTT assay, gelatin zymography, and western blotting were employed. The results showed that Stellettin B significantly inhibited the protein expressions of MMP-2, MMP-9, and uPA, while upregulating the protein expressions of TIMP-1 and TIMP-2. The expressions of p-FAK, p-PI3K, p-AKT, p-mTOR, and MAPKs (p-JNK, p-JUN, p-MAPKp38, and p-ERK) were decreased with increasing concentrations of Stellettin B. Our results suggest that Stellettin B-dependent downregulation of MMP-2 and MMP-9 activities could be mediated by FAK/PI3K/AKT/mTOR and MAPKs signaling pathways in HA22T and HepG2 cells, preventing HCC invasion and migration.

## 1. Introduction

Chemotherapy is an approach used for the systemic treatment of cancer, and plant-derived natural compounds are becoming a focus for the development of new drugs for chemotherapy. For example, Paclitaxel is the most well-known drug isolated from the bark of *Taxus brevifolia* and is used in the treatment of many types of cancer [1]. The mechanism of anticancer drugs is to stop the growth of cancer cells or to destroy them directly, so that the cancer cells undergo a process termed programmed cell death or apoptosis [2].

Marine organisms harbor many novel compounds with varying bioactivities, including anticancer activity. In recent years, research has shifted to the investigation of previously-unexplored resources for potential natural anticancer drugs, and a number of scientists have moved their attention from terrestrial to marine resources. Many marine natural compounds have been shown to inhibit cancer cell proliferation or induce apoptosis through certain cell signaling pathways [3]. Ting et al. [4] isolated Stellettin B from the marine sponge *Jaspis stellifera*, and showed that it has a great inhibitory effect on the growth of human glioblastoma SF295 cells, with low toxicity towards normal cells. The cytotoxic effect of Stellettin B on cancer cells has been demonstrated to act through the PI3K/Akt pathway to inhibit cancer cell proliferation and induce apoptosis. Similarly, Stellettin B was found to cause G1-phase cell-cycle arrest, apoptosis, and autophagy by inhibiting the PI3K/Akt/mTOR pathway [5].

Hepatocellular carcinoma (HCC) is a primary liver cancer and one of the most malignant cancers in many countries, occurring predominantly in men. Surgical removal has long been the mainstay curative treatment for patients with HCC, though this is only feasible in fewer than 20% of cases owing to local spread of cancer cells. Thus, despite advances in technology related to the diagnosis and treatment of this cancer, the incidence of tumor recurrence after resection is still high, and the incidence and morbidity rates of HCC continue to rise. For example, HCC ranks ninth in causes of cancer-related death in the US [6]. Although the cause of HCC is still not completely clear, factors that may increase the risk of HCC are the presence of hepatitis B or C, cirrhosis, high alcohol consumption, diabetes, obesity, excessive iron storage in the liver, and aflatoxin-contaminated food [7]. Chiang et al. [8] isolated bromovulone III, a marine prostaglandin, from soft coral, and showed that it induced apoptosis in hepatocellular carcinoma Hep3B cells through a mechanism that induces ER stress and leads to CHOP/GADD153 and caspase-12 activation. Lin et al. [9] isolated active compound 11-epi-sinulariolide acetate from soft coral, and showed that it prevented the mechanisms of migration and invasion of HCC via inhibition of phosphorylation of ERK1/2 and p38MAPK, and inhibition of the FAK/PI3K/AKT/mTOR pathway.

**Figure 1:**
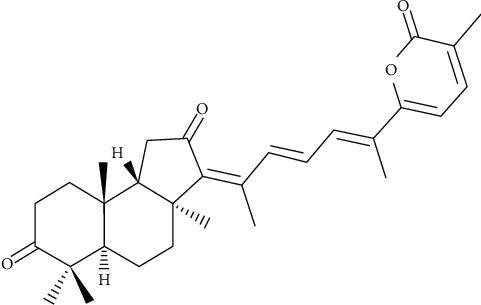
Structure of Stellettin B.

In this study, we isolated Stellettin B from *Stelletta* sp. (Figure 1) and investigated its activities against cell migration and invasion of HCC. HA22T and HepG2 cells were used to evaluate the effects of this compound and study the underlying mechanisms. The results may provide useful information pertaining to the development of new anticancer drugs for the treatment of HCC.

## 2. Materials and Methods

### 2.1. Reagents

Dulbecco's modified Eagle's medium (DMEM), fetal bovine serum (FBS), penicillin, and streptomycin were obtained from Gibco BRL (Grand Island, NY, USA). Stellettin B was extracted from *Stelletta* sp. and dissolved in dimethyl sulfoxide (DMSO). Rabbit anti-human *β*-actin antibody, 3-(4,5-dimethylthiazol-2-yl)-2,5-diphenyltetrazolium bromide (MTT), and other general chemicals were obtained from Sigma-Aldrich Corporation (St Louis, MO, USA). Goat anti-rabbit IgG-conjugated horseradish peroxidase was purchased from EMD Millipore (Billerica, MA, USA). The chemiluminescent substrate for horseradish peroxidas (HRP) western development was obtained from Pierce (Rockford, IL, USA). Rabbit antibodies against human MKK3, MEKK7, GRB2, FAK, mTOR, *p*-mTOR, and RhoA were obtained from Epitomics Inc. (Burlingame, CA, USA). Rabbit antibodies against human TIMP-1 and TIMP-2 were purchased from ProteinTech Group Inc. (Rosemont, IL, USA). Rabbit antibodies against human MMP-2, MMP-9, uPA, PI3K, and *p*-PI3K were obtained from Cell Signaling Technology Inc. (Danvers, MA, USA).

### 2.2. Cell Culture

Hepatocellular carcinoma HA22T and HepG2 cells, purchased from the Taiwan Food Industry Research and Development Institute (Hsinchu, Taiwan), were grown at 37°C in 5% CO_2_ in DMEM containing 10% (*v*/*v*) FBS, 100 units/mL penicillin, and 100 *μ*g/mL streptomycin. When examining the effects of Stellettin B, cells were treated with 10 *μ*L DMSO as a control or with various concentrations of the compound and incubated for 24 h before harvesting for further analyses.

### 2.3. MTT Assay

Cell viability was assessed using the MTT assay [10]. To determine whether the treatment drug was cytotoxic, cells placed in 24-well plates were treated with Stellettin B (6, 12, 18, 24, or 32 *μ*M). After 24 h of incubation, 50 *μ*L of MTT (1 mg/mL in PBS) were added for 3 h, then the culture medium was removed and the cells dissolved in DMSO followed by shaking for 10 min. The plates were then analyzed using a microplate reader (Bio-Rad; Hercules, CA, USA). All of the experiments were repeated three times.

### 2.4. Cell Migration and Invasion Assays

In the cell migration assay, as described previously by Neoh et al. [11], cells were placed into a Boyden chamber (Neuro Probe; Cabin John, MD, USA) at 5 × 10^4^ cells in serum-free medium and treated with or without Stellettin B, followed by incubation for 12 h at 37°C to allow migratory cells to pass through the membrane. In the cell invasion assay, as described previously by Yeh et al. [12], the cells were seeded onto Transwell inserts with 8 *μ*m-pore-size polycarbonate membrane filters coated with 0.5 mg/mL Matrigel; a cell suspension was placed into the upper chamber with serum-free medium, and the bottom chamber was filled with cell-culture medium supplemented with serum. At the end of the incubation period, the membranes coated with migrated and invaded cells in the lower chamber were fixed with methanol and stained with 5% Gimmsa (Merck; Germany); the cells were then counted under a light microscope.

### 2.5. Determination of MMP-2/-9 Activities by Gelatin Zymography

The activities of MMP-2/-9 in conditioned media were detected using gelatin zymography, as described previously by Chen et al. [13]. Cells were incubated for 24 h with different concentrations (0, 6, 12, 18, and 24 *μ*M) of Stellettin B in culture medium, and the collected medium was then concentrated using a speed vacuum. The samples were separated using 10% SDS-PAGE containing 0.2% gelatin under nonreducing conditions, and following separation, the gels were washed three times in wash buffer (100 mM NaCl, 2.5% Triton X-100, 50 mM Tris-HCl, pH 7.5) then activated in reaction buffer (200 mM NaCl, 1 mM CaCl_2_, 0.02% NaN_3_, 1 *μ*M ZnCl_2_, 2% Triton-X 100, 50 mM Tris-HCl buffer, pH = 7.5) for 24 h at 37°C. Finally, the gels were stained with Coomassie Blue R-250, and then destained, and the activities of MMP-2/-9 were quantified using Image J software (NIH, MD, USA).

### 2.6. Western Blotting Analysis

HA22T and HepG2 cells were treated with different concentrations of Stellettin B (0, 6, 12,18, and 24 *μ*M) for 24 h. After stimulation, cells were rinsed twice with ice-cold PBS and 100 *μ*L of cell lysis buffer (20 mM Tris-HCl pH 7.5, 125 mM NaCl, 1% Triton X-100, 1 mM MgCl_2_, 25 mM *β*-glycerophosphate, 50 mM NaF, 100 *μ*M Na_3_VO_4_, 1 mM PMSF, 10 *μ*g/mL leupeptin, and 10 *μ*g/mL aprotinin) were then added to each well. Proteins were denatured in SDS, electrophoresed on 10% SDS-PAGE, and transferred onto a PVDF membrane. Nonspecific binding was blocked with TBST (50 mM Tris-HCl pH 7.5, 150 mM NaCl, and 0.1% Tween 20) containing 5% nonfat milk for 1 h at room temperature. After incubation with the appropriate first antibodies, membranes were washed three times with TBST, and incubated with a secondary antibody for 1 h. After three washes with TBST, the protein bands were detected using the ECL reagent.

### 2.7. Statistical Evaluation

Values are expressed as the mean ± S.E.M. of at least three experiments, which were performed in duplicate. Analysis of variance (ANOVA) was used to assess the statistical significance of differences, and *p* < 0.05 was considered to indicate statistical significance.

## 3. Results

### 3.1. Cytotoxic Effects of Stellettin B on HA22T and HepG2 Hepatoma Cells

**Figure 2:**
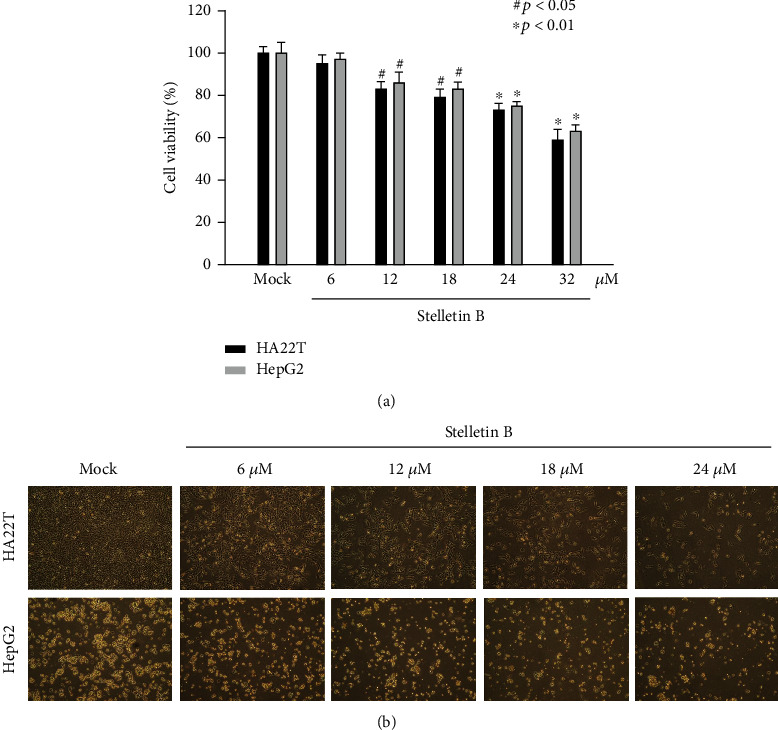
Cytotoxic effects of Stellettin B on HA22T and HepG2 cells. (a) Investigation of the cytotoxic effects of Stellettin B on human hepatoma cell lines. HA22T and HepG2 cells were treated with Stellettin B at concentrations of 6, 12, 18, 24, and 32 *μ*M for 24 h, and an MTT assay was employed to examine the cell viability. The results shown here are representative of three independent experiments (^#^*p* < 0.05; ^∗^*p* < 0.01). (b) Effect of Stellettin B on the morphological changes of HA22T and HepG2 cells treated with Stellettin B at concentrations of 6, 12, 18, and 24 *μ*M for 24 h. (100 × magnification).

The effects of Stellettin B on the cytotoxicity of HA22T and HepG2 hepatoma cells were analyzed using an MTT assay. The test concentrations of Stellettin B were 6, 12, 18, 24, and 32 *μ*M. At a concentration of 24 *μ*M, Stellettin B significantly reduced the survival rates of HA22T and HepG2 cells, to 74% and 76%, respectively. The results demonstrated a dose-response inhibition effect that was positively correlated with the Stellettin B concentration, as shown in Figure 2(a). In order to avoid a low cell survival rate caused by a high drug concentration, a follow-up study was conducted using the concentration range of 6 to 24 *μ*M. Images obtained using a microscope further supported the findings of the MTT assay, showing that Stellettin B suppressed HA22T and HepG2 cell growth at higher doses (Figure 2(b)).

### 3.2. Inhibitory Effects of Stellettin B on Cell Migration and Invasion

**Figure 3:**
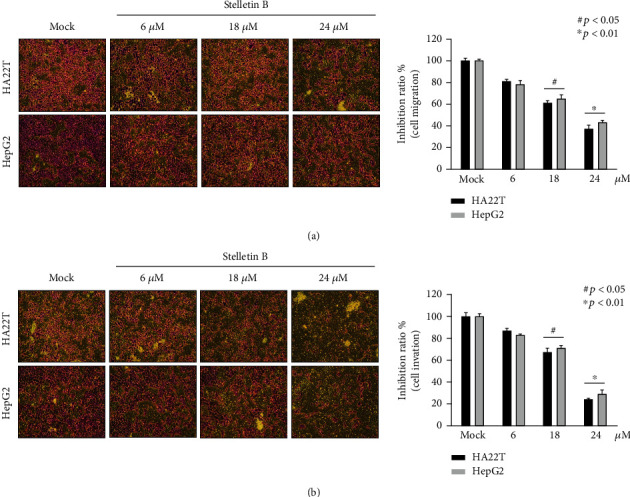
Stellettin B inhibited HA22T and HepG2 cell migration and invasion. (a) Stellettin B inhibited HA22T and HepG2 cell migration; (b) Stellettin B inhibited HA22T and HepG2 cell invasion. Mock refers to cells treated with DMSO vehicle as a control (*n* = 3; three independent experiments). (100 × magnification).

Cell migration and invasion are important processes for the metastasis of cancer cells. In this study, we used a Boyden chamber to analyze the effects of Stellettin B concentrations of 6, 18, 24 *μ*M for 12 h on the migration and invasion of HA22T and HepG2 cells. In terms of cell migration, our results showed that migration of HA22T and HepG2 cells was significantly inhibited with increasing concentrations of Stellettin B, revealing a dose-dependent response (Figure 3(a)). At the concentration of 24 *μ*M, Stellettin B reduced the migration of HA22T and HepG2 cells to 38% and 42%, respectively, suggesting that Stellettin B has good suppression effect on hepatocellular carcinoma cell migration. In terms of cell invasion, a positive correlation between the concentration of Stellettin B and the inhibition effect on cell invasion was observed (Figure 3(b)). At the concentration of 24 *μ*M, Stellettin B reduced the invasion of HA22T and HepG2 cells to 23% and 29%, respectively.

### 3.3. Effects of Stellettin B on the Expression Levels of MMP-2, MMP-9, uPA, TIMP-1, and TIMP-2

**Figure 4:**
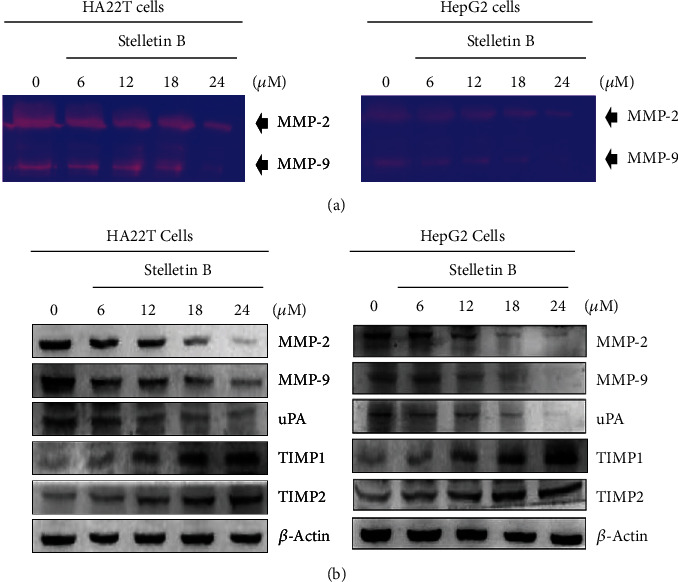
Stellettin B reduced the expressions of MMP-2, MMP-9, and uPA, and increased the expressions of TIMP-1 and TIMP-2. HA22T and HepG2 cells were treated with different concentrations of Stellettin B (0, 6, 12,18, 24 *μ*M) for 24 h, and conditioned media and cell lysates were collected for analysis. (a) Examination of MMP-2/-9 activities by gelatin zymography. (b) The expression levels of MMP-2/-9, uPA, and TIMP-1/-2 were analyzed by western blotting of total cell lysates of HA22T and HepG2 cells treated with Stellettin B. Zero refers to cells treated with DMSO vehicle as a control. *β*-Actin was used as the internal control.

MMP-2 and MMP-9 are members of the matrix metalloproteinase (MMP) family, and can cleave collagen and destroy the extracellular matrix (ECM). Both MMP-2 and MMP-9 are known to be associated with tumor metastasis and angiogenesis. We used gelatin zymography to measure the enzymatic activities of MMP-2 and MMP-9 in HA22T and HepG2 cells treated with Stellettin B. As shown in Figure 4(a), cells treated with different concentrations (6-24 *μ*M) of Stellettin B exhibited decreased hydrolytic activities of MMP-2 and MMP-9, and the decrease was correlated with an increasing concentration of Stellettin B. Furthermore, western blotting was used to analyze the expressions of cell migration- and invasion-related proteins, including MMP-2/-9, uPA, and TIMP-1/-2 (Figure 4(b)). The results showed that contrary to the expressions of MMP-2/-9 and uPA, Stellettin B treatment increased the expressions of TIMP-1 and TIMP-2 proteins. It was therefore revealed that the decreases in cancer cell migration and invasion caused by Stellettin B are associated with downregulation of the activities and protein expressions of MMP-2 and MMP-9.

### 3.4. Effects of Stellettin B on the PI3K/AKT/mTOR Signaling Pathway

**Figure 5:**
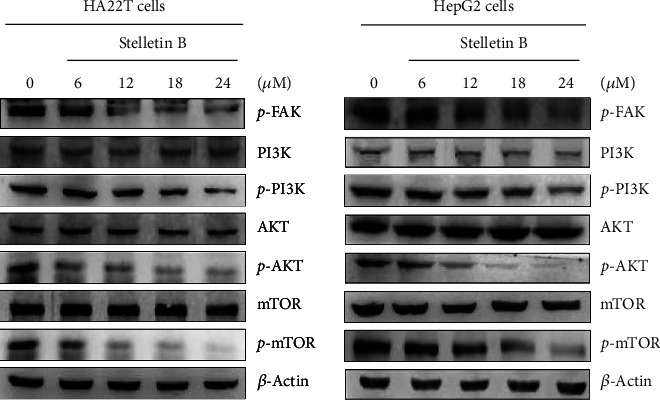
Stellettin B regulated the expression levels in the PI3K/AKT/mTOR signaling pathway. Western blotting analysis was employed to analyze the expressions levels of *p*-FAK, *p*-PI3K, *p*-AKT, and *p*-mTOR in HA22T and HepG2 cells treated with Stellettin B. Zero refers to cells treated with DMSO vehicle as a control. *β*-Actin was used as the internal control.

The PI3K/AKT/mTOR signaling pathway is involved in cell proliferation, differentiation, survival, and metastasis [14]. In this study, we investigated whether the inhibition effects of Stellettin B on cell migration and invasion of HA22T and HepG2 cells are mediated by regulation of the PI3K/Akt/mTOR pathway. As shown in Figure 5, phosphorylated FAK, PI3K, AKT, and mTOR were decreased with an increasing Stellettin B concentration, while the PI3K, AKT, and mTOR levels remained unchanged. These results suggested that Stellettin B may inhibit HA22T and HepG2 cell migration and invasion through blocking the phosphorylation of the FAK/PI3K/Akt/mTOR pathway.

### 3.5. Effects of Stellettin B on the Expressions of Cell Migration- and Invasion-Related Proteins

**Figure 6:**
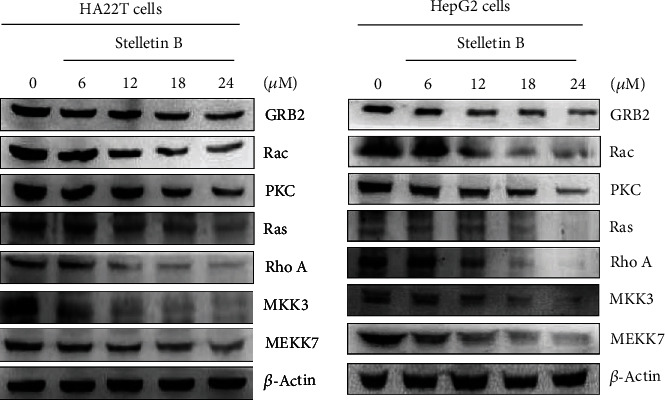
Stellettin B suppressed the expression levels of proteins associated with cell migration and invasion. Western blotting analysis was used to examine the expression levels of RhoA, Ras, Rac, PKC, GRB2, MKK3, and MEKK7 in HA22T and HepG2 cells treated with Stellettin B. Zero refers to cells treated with DMSO vehicle as a control. *β*-Actin was used as the internal control.

We further studied the detailed molecular mechanism of Stellettin B by measuring changes in cell migration- and invasion-related proteins, including RhoA, Ras, PKC, growth factor receptor-bound protein 2 (GRB2), mitogen-activated protein kinase kinase 3 (MKK3), and MAP kinase kinase kinase 7 (MEKK7). As presented in Figure 6, western blotting showed that Stellettin B downregulated the expressions of these cell migration- and invasion-related proteins in both HA22T and HepG2 cells.

### 3.6. Effects of Stellettin B on the Expressions of MAPKs Pathway-Related Proteins

**Figure 7:**
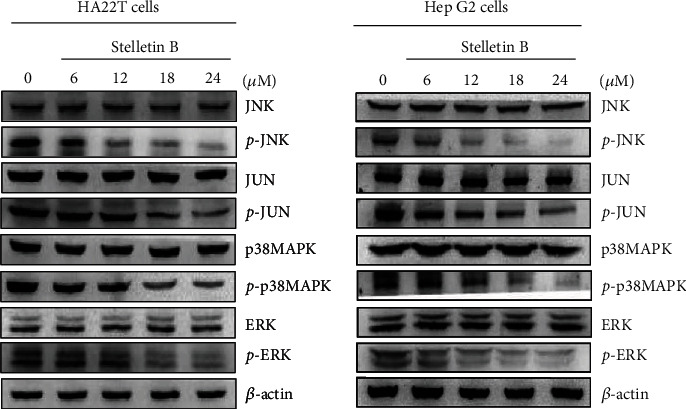
Effects of Stellettin B on the MAPKs signaling pathway. HA22T and HepG2 cells were treated with different concentrations of Stellettin B (0, 6, 12, 18, and 24 *μ*g/mL) and cell lysates were collected for western blotting analysis. The MAPKs-related proteins were validated, including p38MAPK, *p*-p38MAPK, ERK, *p*-ERK, JNK, *p*-JNK, JUN, and *p*-JUN. Zero refers to cells treated with DMSO vehicle as a control. *β*-Actin was used as the internal control.

Mitogen-activated protein kinases (MAPKs) play important roles in the regulation of cell proliferation, differentiation, migration, senescence, and apoptosis [15]. Wu et al. [16] found that sinulariolide, a compound obtained from marine soft coral, inhibited MMP-2/-9 activities through downregulating MAPK, and thus inhibited the migration and invasion abilities of HA22T cells. Therefore, in this study, we investigated the relationships of cell migration and invasion with the MAPKs pathway in Stellettin B-treated HA22T and HepG2 cells. The western blotting results showed that Stellettin B inhibited the expressions of *p*-JNK, *p*-JUN, *p*-MAPKp38, and *p*-ERK in HA22T and HepG2 cells (Figure 7).

## 4. Discussion

Once anticancer drugs enter the body, they induce cancer-cell death by causing cellular events that inhibit cell growth or lead to apoptosis or necrosis. Evaluation of the cytotoxicity of a chemotherapy agent assesses their effects on cell morphology, such as the shape of the cells and damage to the cell membrane. Inhibition of cell growth is also an excellent index by which to measure the efficacy of anticancer drugs. Our results showed that Stellettin B had cell cytotoxic and inhibitory effects on cell migration in HA22T and HepG2 cells (Figures 1–2), which suggested that Stellettin B is more effective in human HCC cells and could be a potential therapeutic drug for the treatment of metastatic HCC cells.

The MTT assay is a simple and direct measurement of cell growth; this assay was employed in the present study to examine the cytotoxicity of Stellettin B towards HA22T and HepG2 cells, and showed significant inhibition of cell growth at 24 *μ*M, reducing the cell survival rates to 74% and 76%, respectively (Figure 2(a)). Changes in the pattern of cell growth and increased suppression of cell proliferation were also observed with an increasing Stellettin B concentration (Figure 2(b)).

Metastasis is the main cause of death of patients with cancer. Cheng et al. [17] isolated sinulariolide from coral, and showed that it could inhibit cell migration and invasion of human bladder carcinoma TSGH-8301 cell line by decreasing the expressions of MMP-2/MMP-9 and urokinase and increasing the expressions of TIMP-1/TIMP-2. Later, Cheng and colleagues [18] revealed for the first time that marine sponge derivative Stellettin B has anti-invasion and anti-angiogenic effects on glioblastoma cells. In this study, we investigated the cytotoxicity of Stellettin B towards hepatocellular carcinoma, and found that at 18 *μ*M it reduced cell migration and invasion to 58-63%, demonstrating that *Stelletta* sp. also contains a compound with an anticancer property (Figure 3).

MMPs are calcium-dependent zinc-containing endopepidases; they are involved in the breakdown of the extracellular matrix (ECM), and are important enzymes associated with invasion of cancer cells [19]. Among the members of MMP family proteins, MMP-2 and MMP-9 are considered to play important roles in the metastasis of tumor cells [20]. Huang et al. [21] found that beta-mangostin could inhibit the migration and invasion of human HCC cells by reducing the expressions and activities of MMP-2 and MMP-9, and the process involved the activation of the MEK1/2, ERK1/2, MEK4, and JNK1/2 signaling pathways.

Tissue inhibitor of metalloproteinases (TIMPs) are endogenous inhibitors of MMPs, and therefore dynamic changes between MMPs and TIMPs can be used to detect effects on tumor cell metastasis [22]. Urokinase, also known as urokinase-type plasminogen activator (uPA) [23], is a serine protease found in the ECM of many tissues, and is mainly responsible for the cleavage of plasminogen into plasmin, which further degrades the ECM. Neoh et al. [24] found that flaccidoxide-13-acetate, a compound obtained from soft coral *Cladiella kashmani*, reduced the migration and invasion of bladder cancer cells by inhibiting the expressions of MMP-2, MMP-9, and uPAR, and increasing the expressions of TIMP-1 and TIMP-2 proteins, which are mediated by regulating the FAK/PI3K/AKT/mTOR pathway. Additionally, Wu et al. [25] isolated flaccidoxide-13-acetate from soft coral *Sinularia gibberosa,* and found that it induced apoptosis in bladder cancer cells by reducing the expressions of MMP-2, MMP-9, and uPA and increasing those of TIMP-1 and TIMP-2. In the present study, we found that the activities and expressions of MMP-2, MMP-9, and uPA were significantly inhibited by Stellettin B treatment, and the expressions of TIMP-1 and TIMP-2 were increased (Figure 4). The results indicated that Stellettin B can inhibit the metastatic activity of hepatocellular carcinoma cells.

The PI3K/AKT/mTOR pathway is known to be involved in cell proliferation, cell differentiation, cell survival, and metastasis. Wu et al. [26] showed that sinulariolide could inhibit gastric cancer cell migration and invasion through reduction of the activities and performance of MMP-2 and MMP-9, which are mediated by the FAK/PI3K/AKT signaling pathway. Yang et al. [27] also reported that bornyl cis-4-hydroxycinnamate suppressed cell metastasis of melanoma by reducing the expressions of MMP-2 and MMP-9 through the FAK/PI3K/Akt/mTOR pathway [28, 29]. Similarly, in this study, we investigated the effects of Stellettin B on cell migration and invasion of HA22T and HepG2 cells, and found that the expressions of *p*-FAK, *p*-PI3K, *p*-AKT, and *p*-mTOR were decreased with an increasing Stellettin B concentration. Our results suggested that the FAK/PI3K/AKT/mTOR pathway affects the activities and expressions of MMP-2 and MMP-9, further inhibiting the migration and invasion of hepatocellular carcinoma cells (Figure 5).

In addition, the results of this study showed that Stellettin B downregulates the expressions of several cell migration- and invasion-associated proteins (Figure 6). RhoA and Rac belong to the small GTPase Rho protein family [30, 31], and are involved in cell attachment, movement, and growth. RAS is involved in tumor formation and growth [32], and PKC is closely related to cell growth, apoptosis, and migration, and is considered a cancer promoter [33]. GRB2 plays an important role in activating the downstream signaling pathway [34]. MKK3 is an upstream MAPK kinase that receives stimulation from multiple extracellular signaling and activates downstream pathways [35], while MEKK7 is an important kinase that regulates MAPK signaling [36]. Our western blotting analysis indicated that Stellettin B downregulated the expressions and activities of *p*-JNK, *p*-JUN, *p*-p38MAPK, and *p*-ERK in HA22T and HepG2 cells, suggesting that its effects are mediated by the MEKK7/MKK3/MAPKs pathway (Figure 7).

## 5. Conclusion

**Figure 8:**
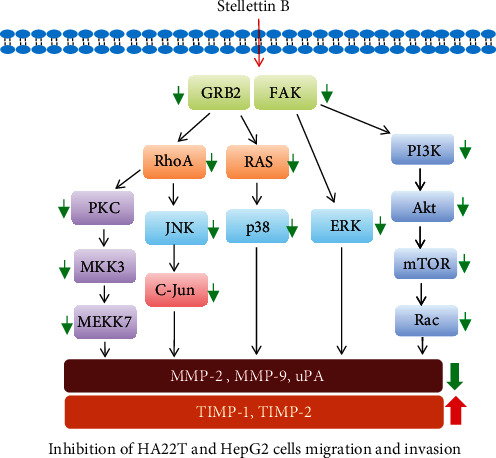
Hypothetical illustration of the Stellettin B associated pathway in HA22T and HepG2 cells.

The causes of cancer development have been widely investigated, but effective treatments for many types of cancer are still being sought. Radiotherapy remains one of the most common approaches for the treatment of cancer, but it is not the best option for metastatic cancers. The hypothetical mechanism of Stellettin B in HA22T and HepG2 human HCC cells is illustrated in Figure 8. The results of the current study indicated that Stellettin B has the potential to be developed as an effective chemotherapeutic agent for metastatic cancers such as HCC.

## Data Availability

The data used to support the findings of this study are included within the article.
